# Proteomic analysis reveals large amounts of decomposition enzymes and major metabolic pathways involved in algicidal process of *Trametes versicolor* F21a

**DOI:** 10.1038/s41598-017-04251-1

**Published:** 2017-06-20

**Authors:** Xueyan Gao, Congyan Wang, Wei Dai, Shenrong Ren, Fang Tao, Xingbing He, Guomin Han, Wei Wang

**Affiliations:** 1 School of Life Sciences, Anhui Agricultural University, Hefei, 230036 China; 2 Institute of Environment and Ecology, Academy of Environmental Health and Ecological Security & School of the Environment and Safety Engineering, Jiangsu University, Zhenjiang, 212013 China; 3 College of Biology and Environmental Sciences, Jishou University, Jishou, 416000 China

## Abstract

A recent algicidal mode indicates that fungal mycelia can wrap and eliminate almost all co-cultivated algal cells within a short time span. However, the underlying molecular mechanism is rarely understood. We applied proteomic analysis to investigate the algicidal process of *Trametes versicolor* F21a and identified 3,754 fungal proteins. Of these, 30 fungal enzymes with endo- or exoglycosidase activities such as *β*-1,3-glucanase, *α*-galactosidase, *α*-glucosidase, alginate lyase and chondroitin lyase were significantly up-regulated. These proteins belong to Glycoside Hydrolases, Auxiliary Activities, Carbohydrate Esterases and Polysaccharide Lyases, suggesting that these enzymes may degrade lipopolysaccharides, peptidoglycans and alginic acid of algal cells. Additionally, peptidase, exonuclease, manganese peroxidase and cytochrome c peroxidase, which decompose proteins and DNA or convert other small molecules of algal cells, could be other major decomposition enzymes. Gene Ontology and KEGG pathway enrichment analysis demonstrated that pyruvate metabolism and tricarboxylic acid cycle pathways play a critical role in response to adverse environment via increasing energy production to synthesize lytic enzymes or uptake molecules. Carbon metabolism, selenocompound metabolism, sulfur assimilation and metabolism, as well as several amino acid biosynthesis pathways could play vital roles in the synthesis of nutrients required by fungal mycelia.

## Introduction

Blue algae (Blue-green algae or Cyanobacteria) belong to a phylum of bacteria, and can produce energy by photosynthesis^1^. Most members possess nitrogen fixation capacity and resistance to adverse environments^2, 3^. Although blue algae produce most of the microbial biomass for primary consumers in waterbodies, the rapid growth of cyanobacteria often results in algal blooms in lakes under eutrophication^4, 5^. Algal blooms outbreaks have significantly negative impacts on ecosystems as well as on human beings, such as reduced transparency and dissolved oxygen, generation of stench, and threat to aquatic biological safety^6–9^. Thus, it is extremely urgent to establish an efficient method for controlling the incidence of algal blooms and their toxic metabolites. Currently, three main approaches are used to control algal blooms involving chemical strategies, physical methods and biomanipulation^10^. Chemical strategies are prone to generate secondary pollution in the environment^11^. Physical treatments are suitable for short-term solutions, but cannot completely eliminate the blooms^11^. In contrast, biomanipulation is considered to be a cost-effective and safe approach to eliminate algal blooms^7^.

The accumulated evidences indicate that several bacteria and fungi can inhibit algal growth or degrade algal cells^4^. Numerous investigations have mainly focused on algicidal bacteria, while fungi with algicidal potential have been less well studied^8^. The antagonistic activities of 62 fungal isolates toward cyanobacteria were first observed in 1978. The cephalosporin group of antibiotics was deemed to be important algicidal factors released by *Acremonium* and *Emericellopsis* spp.^12^. In addition, a novel type of algicidal fungus was first identified owing to its ability to lyse live blue algae^7^. Mycelia of *Trichaptum abietinum* 1302BG can wrap algal cells and remove most algae within 48 h, resulting in clarification of the algal solution. Similar algicidal properties were also observed in the fungus *L*. *spadicea* ^8^. We had earlier identified *Trametes versicolor* F21a, *Bjerkandera adusta* T1, *T*. *hirsuta* T24, and *Irpex lacteus* T2b to have strong algicidal abilities. Of these, *T*. *versicolor* F21a showed the strongest algicidal ability by eliminating almost all tested living algal cells within 30 h^11^.

Several aspects of the underlying algicidal mechanism of these fungi were investigated at the cellular and biochemical levels. The interaction between fungus *T*. *abietinum* 1302BG and algal cells was investigated by scanning electron microscopy and transmission electron microscopy^7^. The hyphae of *T*. *abietinum* 1302BG could initially recognize the algal cells, and then wrap and degrade them^7^. Flow cytometry and fourier transform infrared spectroscopy were also employed to assess and compare the cell and surface morphology of the fungus *Phanerochaete chrysosporium* ^10^, revealing that *P*. *chrysosporium* primarily destroyed the cell membrane, cell wall and subsequently the pyridine ring of chlorophyll. Preliminary investigation of a few extracellular enzymes of *T*. *versicolor* F21a during the algicidal process indicated that the activities of cellulase, *β*-glucosidase, protease, alkaline phosphatase, laccase and manganese peroxidase were significantly induced in different phases of fungal mycelia^13^. The numbers and types of enzymes with decomposition abilities are far greater than the number of enzymes tested. For example, comparative analyses of 31 fungal genomes showed that as many as 250 genes are candidates for decomposition of lignin and cellulose^14^. Until now, it is still not known how many types of decomposition enzymes are involved in the complex algicidal process.

In this study, proteomic analysis was used to detect and quantify the whole proteins during the algicidal process of the fungus *T*. *versicolor* F21a. The aims of the present study were: (1) to use an approach combining TMT labeling and LC-MS/MS to reveal the whole proteins of *T*. *versicolor* F21a during the algicidal process; (2) to reveal the types of fungal decomposition enzymes and main metabolism pathways during the algicidal process. Our results will provide detailed information on the expressed decomposition enzymes as well as the major metabolism pathways operating during the algicidal process, and will be helpful in further understanding the underlying molecular mechanism of this novel fungi-algae interaction.

## Results

### Effect of *T*. *versicolor* F21a on chlorophyll-a contents of *M*. *aeruginosa* PCC7806

**Figure 1:**
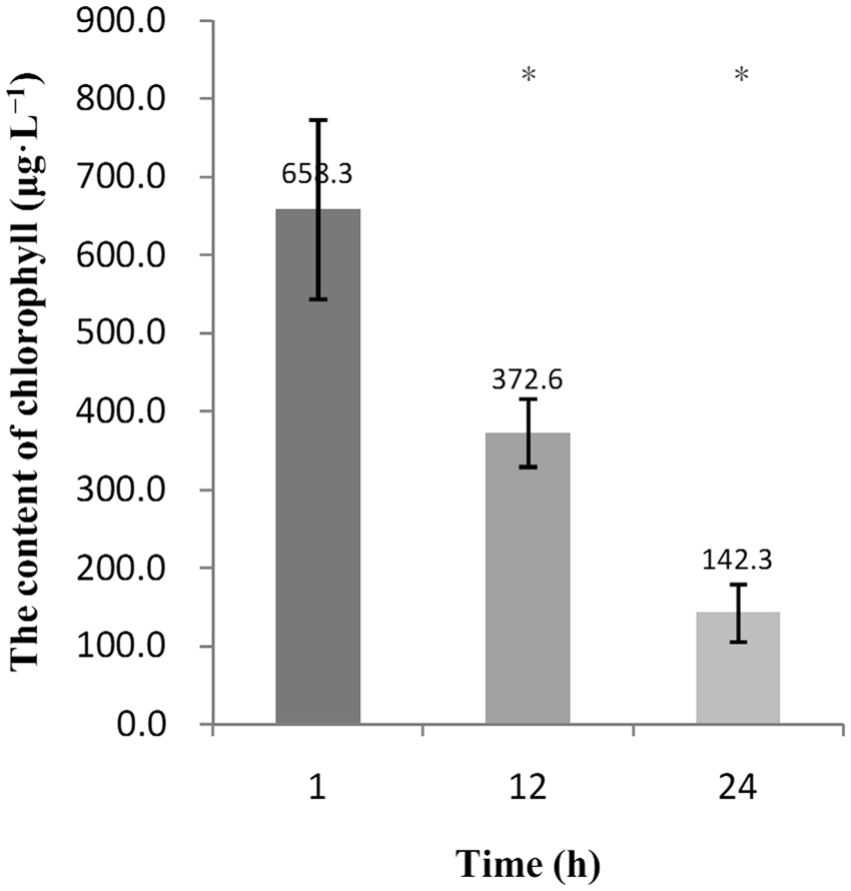
Changes in chlorophyll-a content during the algicidal process of *T*. *versicolor* F21. T12, 12 h fungal mycelia co-cultivated with algal cells; T1, 1 h fungal mycelia co-cultivated with algal cells. Significant differences between the control and treatments are indicated by asterisk (*p* < 0.05).

Growth of *M*. *aeruginosa* PCC7806 was assessed when co-cultivated with mycelia of *T*. *versicolor* F21a. The initial chlorophyll-a content of *M*. *aeruginosa* PCC7806 was 658.3 μg·L^−1^. After co-cultivation for 12 h and 24 h, the chlorophyll-a contents decreased to 372.6 μg·L^−1^ (43.40%) and 142.3 μg·L^−1^ (78.38%), respectively (Fig. 1). The algal media co-cultivated with fungal mycelia turned transparent after 48 h of treatment.

### Identification of proteins related to algicidal activity using TMT LC-MS/MS

**Figure 2:**
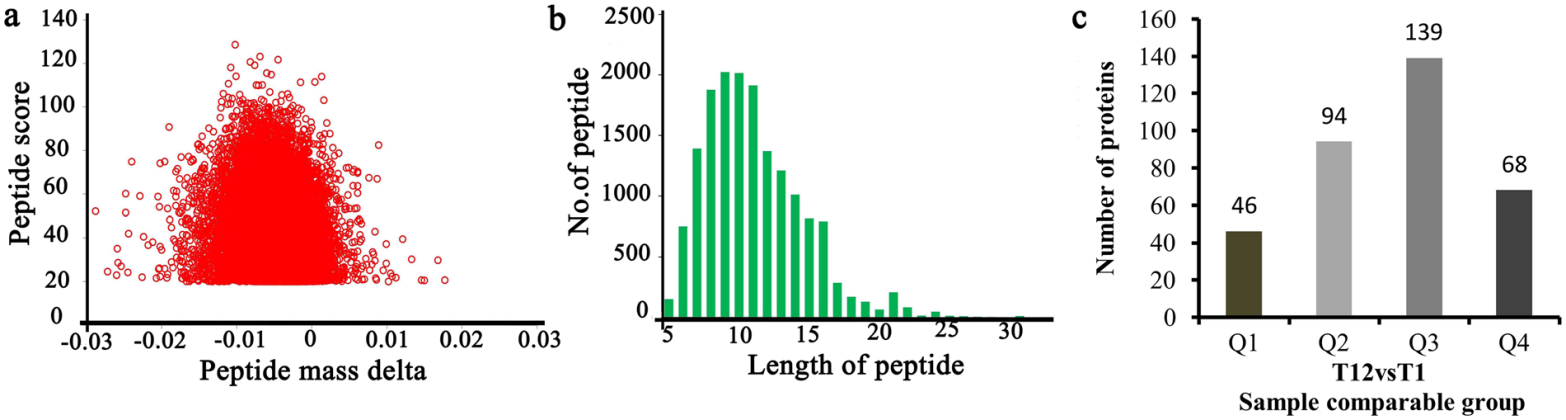
Proteins identified during the algicidal process of *T*. *versicolor* F21a. (**a**) Mass error distribution of the identified peptides, (**b**) Peptide length distribution, (**c**) Distribution of significantly differentially regulated proteins. Q1 (0 < Ratio L/H < 0.67), Q2 (0.67 < Ratio L/H < 0.83), Q3 (1.2 < Ratio L/H < 1.5) and Q4 (Ratio L/H > 1.5), *p* < 0.05.

The draft genome sequence of *T*. *versicolor* FP-101664 SS1 was completed by the Department of Energy Joint Genome Institute (JGI)^14^, which facilitated systematic analysis of proteins related to the algicidal process of *T*. *versicolor* F21. To ensure the quality of proteomic analysis, the generated MS data were assessed. As shown in Fig. 2a, the distribution of mass error was close to zero with most of the values being less than 0.02 Da, indicating mass accuracy of the MS data (Fig. 2a). The length of most peptides was between 8 and 16 (Fig. 2b). Based on the above data, sample preparations were regarded to have reached the standard.

A total of 3,754 proteins were identified by TMT Labeling coupled with LC-MS/MS analysis, among which 2,809 proteins could be quantified. Genes encoding 207 proteins were up-regulated and those encoding 140 proteins were down-regulated in the 12 h fungal samples when compared with those at 1 h (Fig. 2c).

### Functional enrichment of proteins related to algicidal activity

**Figure 3:**
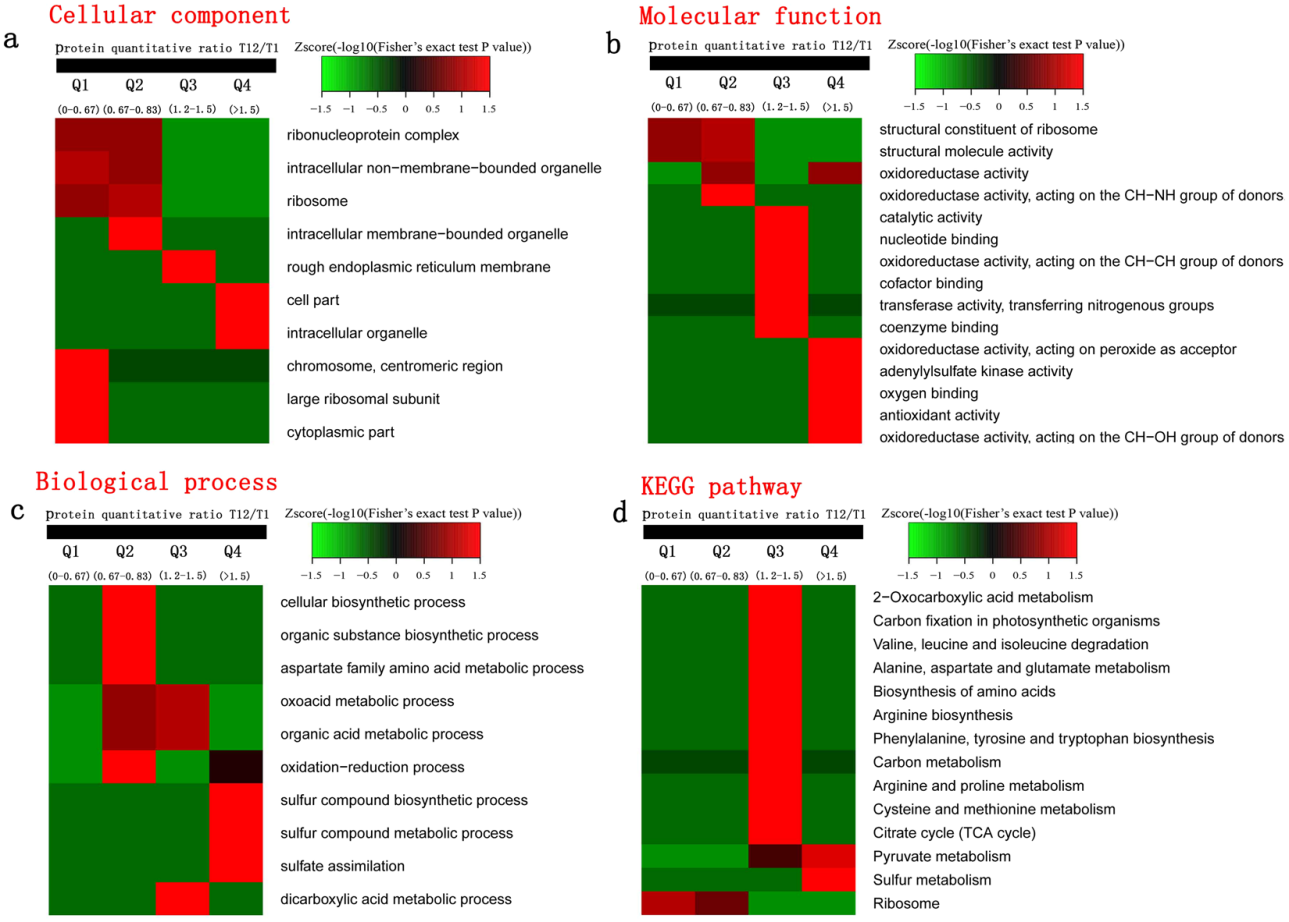
Functional enrichment-based clustering analysis of Quantitative Categories. (**a**) Cellular component, (**b**) Molecular function, (**c**) Biological process, (**d**) KEGG pathway.

Four categories of cellular components, 12 categories of molecular functions and ten categories of biological processes were the enriched Gene Ontology (GO) terms^15^ among all differentially expressed genes (Table 1). Up-regulated proteins in the cellular component category belonged to “cell part” and “intracellular organelle” (Fig. 3a). Up-regulated proteins in the molecular function category belonged to “oxidoreductase activity, acting on peroxide as acceptor”, “oxidoreductase activity acting on the CH-OH group of donors”, “oxygen binding”, “adenylylsulfate kinase activity” and “antioxidant activity” (Fig. 3b). Up-regulated proteins in the biological process category belonged to “sulfur compound metabolic process”, “sulfate assimilation” and “sulfur compound biosynthetic process” (Fig. 3c).

**Table 1:** The GO term enrichment of different proteins.

GO Terms Level 1	GO Terms Description	Mapping	Background	Fisher’ exact test P value	−log10(P value)
Cellular Component	ribosome	10	66	0.007384	2.13
	protein complex	3	168	0.022873	1.64
	intracellular membrane-bounded organelle	8	285	0.028706	1.54
	membrane-bounded organelle	8	285	0.028706	1.54
Molecular Function	oxidoreductase activity	38	361	0.000305	3.52
	structural constituent of ribosome	10	55	0.002324	2.63
	cofactor binding	12	92	0.012495	1.90
	adenylylsulfate kinase activity	2	2	0.016525	1.78
	transferase activity, transferring acyl groups	2	2	0.016525	1.78
	oxidoreductase activity, acting on a sulfur group of donors	3	9	0.024393	1.61
	catalytic activity	104	1560	0.025367	1.60
	structural molecule activity	10	78	0.026482	1.58
	antioxidant activity	4	18	0.028929	1.54
	coenzyme binding	9	69	0.036116	1.44
	oxidoreductase activity, acting on the CH-NH group of donors	2	4	0.038428	1.42
	oxidoreductase activity, acting on the CH-OH group of donors	4	22	0.049980	1.30
Biological Process	dicarboxylic acid metabolic process	4	7	0.002086	2.68
	organic acid biosynthetic process	9	56	0.007818	2.11
	sulfur compound metabolic process	5	19	0.008294	2.08
	sulfur compound biosynthetic process	4	12	0.009284	2.03
	branched-chain amino acid biosynthetic process	3	6	0.010509	1.98
	sulfate assimilation	2	2	0.016525	1.78
	dicarboxylic acid biosynthetic process	2	2	0.016525	1.78
	cellular amino acid biosynthetic process	7	43	0.017344	1.76
	branched-chain amino acid metabolic process	3	8	0.019044	1.72
	aspartate family amino acid metabolic process	3	9	0.024393	1.61

KEGG pathway enrichment analysis revealed that the major enriched pathways were “ribosome”, “carbon metabolism”, “pyruvate metabolism”, “citrate cycle (TCA cycle)”, “glycolysis/gluconeogenesis”, “biosynthesis of amino acids”, etc. (Table 2, Figs S1–S3)^16–18^. KEGG pathway cluster analysis showed that “pyruvate metabolism” and “sulfur metabolism” were significantly up-regulated (Fig. 3d). Particularly, many genes of carbon metabolism, citrate cycle (TCA cycle) pathways, and biosynthesis of amino acids were up-regulated (Figs S1–S3)^16–18^.

**Table 2:** KEGG pathway-based enrichment analysis of different proteins.

KEGG pathway	Mapping	Background	Fisher’ exact test p value	−log10(P value)
Ribosome	18	104	0.000181	3.74
Carbon metabolism	17	106	0.000494	3.31
Pyruvate metabolism	8	38	0.008602	2.07
Pentose and glucuronate interconversions	6	27	0.021394	1.67
Selenocompound metabolism	3	6	0.021394	1.67
Glycolysis/Gluconeogenesis	8	49	0.024284	1.61
Arginine biosynthesis	4	16	0.031222	1.51
Alanine, aspartate and glutamate metabolism	5	24	0.031222	1.51
Carbon fixation in photosynthetic organisms	4	16	0.031222	1.51
2-Oxocarboxylic acid metabolism	6	33	0.031222	1.51
Biosynthesis of amino acids	13	115	0.031222	1.51
Citrate cycle (TCA cycle)	5	29	0.045084	1.35
Phenylalanine metabolism	4	19	0.045084	1.35
Phenylalanine, tyrosine and tryptophan biosynthesis	4	19	0.045084	1.35
Carbon fixation pathways in prokaryotes	3	10	0.045084	1.35

### Classification of subcellular location

**Figure 4:**
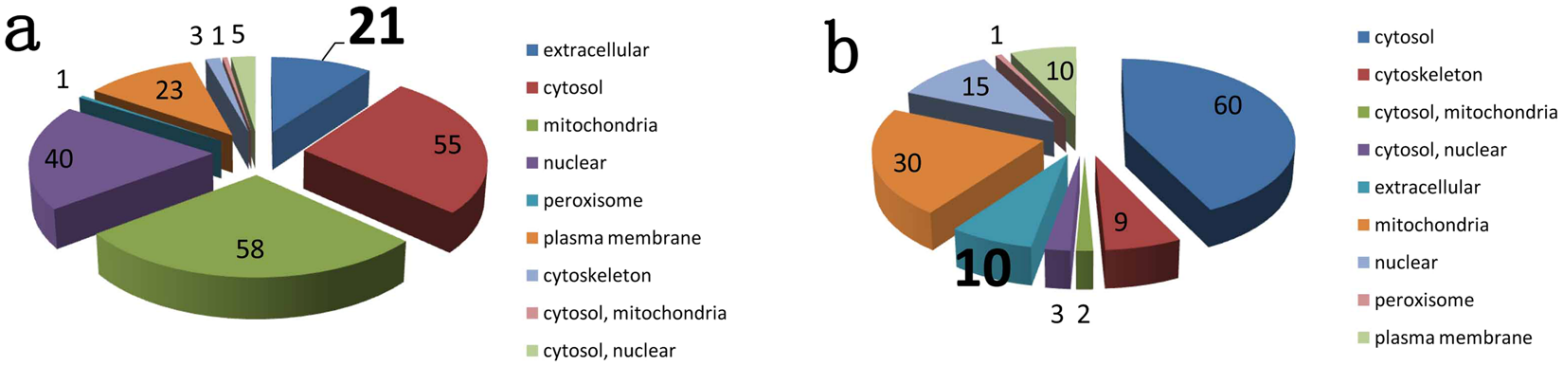
The subcellular locations of up-regulated (**a**) and down-regulated (**b**) proteins.

The significantly up-regulated proteins were mainly located in the cytosol, mitochondria, nucleus, plasma membrane and extracellular space, while the frequently down-regulated proteins were located in the cytosol, mitochondria, nucleus, plasma membrane, and extracellular space (Fig. 4). Remarkably, extracellular proteins comprised ~10% of all significantly up-regulated proteins.

### Domain enrichment-based clustering analysis

**Figure 5:**
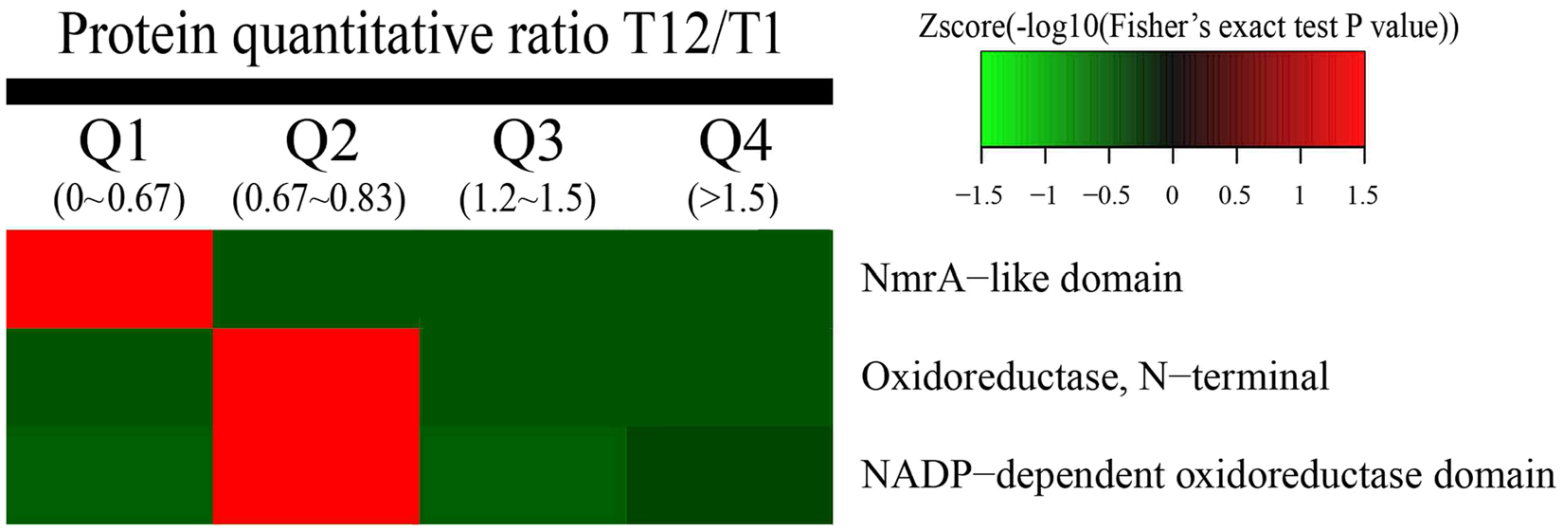
Protein domain enrichment-based clustering analysis of different proteins.

Protein domain enrichment-based clustering analysis of differentially regulated proteins showed that NmrA-like domain and Oxidoreductase domain were the significantly down-regulated fungal proteins during the algicidal process (Fig. 5).

### Biomass degradation enzymes

**Figure 6:**
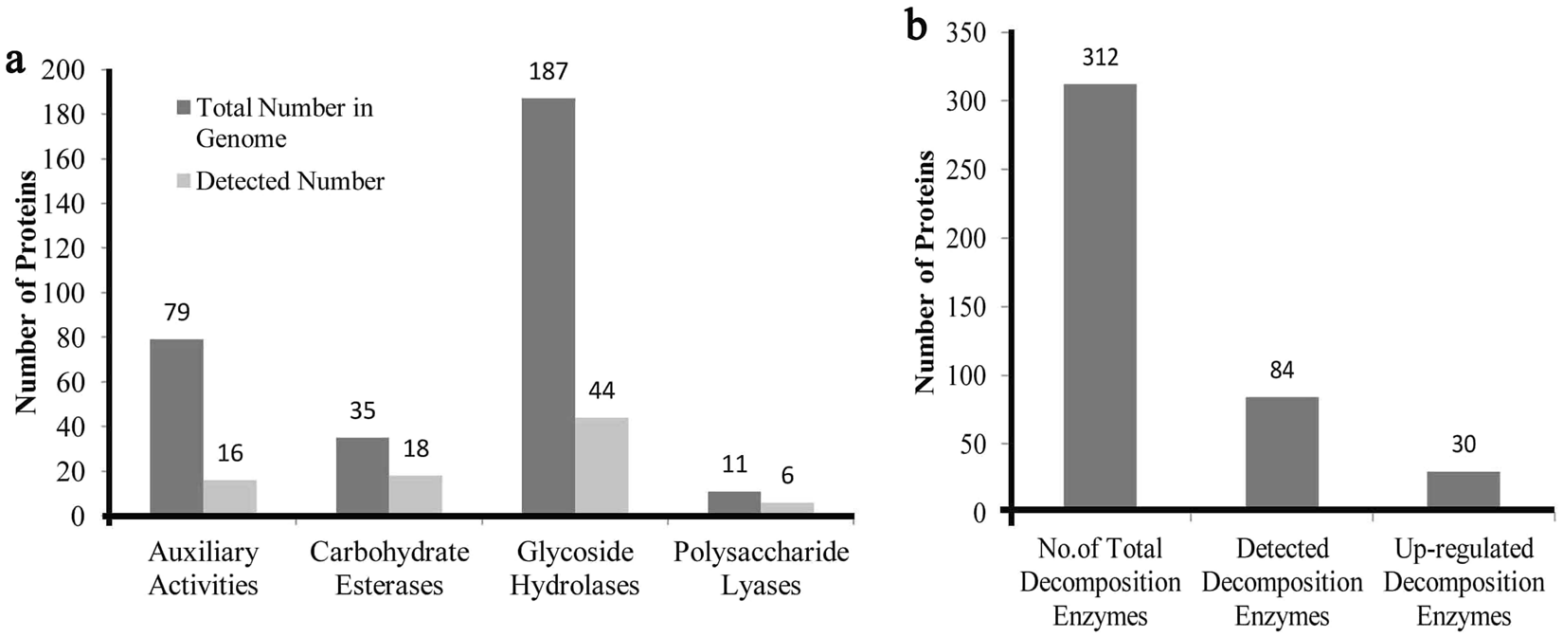
Summary of detected degradation enzymes during the algicidal process of *T*. *versicolor* F21a. (**a**) Classification of detected degradation enzymes, (**b**) Number of detected degradation enzymes.

312 different proteins with biomass degradation capacities were predicted from the *T*. *versicolor* FP-101664 SS1 genome (Fig. 6a). They can be classified into four classes, namely Auxiliary Activities, Carbohydrate Esterases, Glycoside Hydrolases, and Polysaccharide Lyases (Table S1). These enzymes can be further classified into 46 subclasses. Majority of the degradation enzymes belong to Glycoside Hydrolases.

12 h after co-cultivation, 84 of the 312 fungal proteins could be detected by TMT Labeling LC-MS/MS analysis (Fig. 6b, Table S2). The detected proteins were grouped into 31 subclasses (Table S1). 44 out of 187 proteins belonged to Glycoside Hydrolases. More than half of the proteins (18 out of 35, and 6 out of 11) detected consisted of Carbohydrate Esterases and Polysaccharide Lyases. When compared with the 1 h samples, 30 proteins were up-regulated in the fungal mycelia at 12 h (Table 3). Total of 13 up-regulated proteins with quantitative ratios above 1.2, particularly proteins with ID 44897 (manganese peroxidase), 134935 (*β*-1,3-glucanase), 58033 (α-glucosidase) and 111754 (chondroitin ABC lyase) were up-regulated with quantitative ratios above 2 (Table 3). Approximately one third of up-regulated proteins contain secretary signal peptides, while 7 of 30 proteins contain signal peptides that can guide the transportation of the mature protein to the mitochondrion.

**Table 3:** Up-regulated decomposition enzymes of *T*. *versicolor* F21a.

Protein Id	NCBI annotation	CAZymes classification	Location	T12/T1 Ratio	P value	Significant
26239	manganese peroxidase	AA2	S	1.47	0.286007	
26601	cytochrome c peroxidase	AA2	M	1.329	0.005797	yes
**44897**	**manganese peroxidase**	**AA2**	**S**	**3**.**702**	**0**.**004783**	**yes**
174536	glucose-methanol-choline (gmc) oxidoreductase family	AA3	—	1.168	0.289844	
174721	pyranose oxidase, alcohol oxidase	AA3	M	1.264	0.23381	
57372	glucose-methanol-choline (gmc) oxidoreductase family	AA3	S	1.116	0.388914	
59756	alcohol oxidase	AA3	—	1.011	0.895376	
61229	copper radical oxidase, galactose oxidase, glyoxal oxidase	AA5	—	1.95	0.000907	yes
116106	phospholipase carboxylesterase	CE1	M	1.067	0.466033	
27033	3-hydroxyacyl CoA dehydrogenase	CE1	—	1.218	0.039281	yes
171192	*β*-glucosidase	GH1	—	1.187	0.424115	
75497	*α*-1,3-glucan synthase	GH13	S	1.223	0.006005	yes
156418	Six-hairpin glycosidase	GH15	—	1.167	0.015216	yes
**134935**	***β***-**1**,**3**-**glucanase**	**GH16**	—	**2**.**461**	**0**.**004073**	**yes**
137261	glucosidase	GH16	M	1.065	0.409968	
175484	endo-1,3(4)- *β*-glucanase	GH16	S	1.261	0.035876	yes
159574	*α*-galactosidase	GH27	S	1.003	0.98303	
146818	*β*-glucosidase	GH3	—	1.092	0.347247	
173291	*α*-glucosidase	GH31	S	1.167	0.050646	
**58033**	**α**-**glucosidase**	**GH31**	**S**	**2**.**145**	**0**.**00371**	**yes**
123722	Mannosyl-oligosaccharide 1,2-*α*-mannosidase	GH47	M	1.265	0.002694	yes
54985	Mannosyl-oligosaccharide 1,2-*α*-mannosidase	GH47	M	1.119	0.348562	
172368	Glucan 1,3-*β*-glucosidase 3	GH5	—	1.05	0.548148	
32196	glucocerebrosidase	GH5	—	1.131	0.312239	
120979	exo-*β*-1,3-glucanase	GH55	—	1.016	0.937197	
37162	cellobiohydrolase	GH74	S	1.065	0.06072	
34231	alginate lyase	PL14	S	1.136	0.304725	
138905	3-ketoacyl-CoA thiolase 5, peroxisomal	PL15	—	1.214	0.074484	
**111754**	**chondroitin ABC lyase**	**PL8**	**M**	**6**.**881**	**0**.**001413**	**yes**
159259	Chondroitinase-AC	PL8	S	1.06	0.669082	

Location, Prediction of localization; C, Chloroplast; M, Mitochondrion; S, Secretory pathway; -, Any other location; GH, Glycoside Hydrolases; AA, Auxiliary Activities; CE, Carbohydrate Esterases; PL, Polysaccharide Lyases; Changes of more than 1.2 and P values less than 0.05 were considered significant.

Additionally, several fungal proteins belonging to peptidase, exonuclease, manganese peroxidase, cytochrome c peroxidase, glucose-methanol-choline oxidoreductase and alcohol oxidase categories were also up-regulated during the algicidal process (Table S3).

### Enzymatic activities during the algicidal process

**Figure 7:**
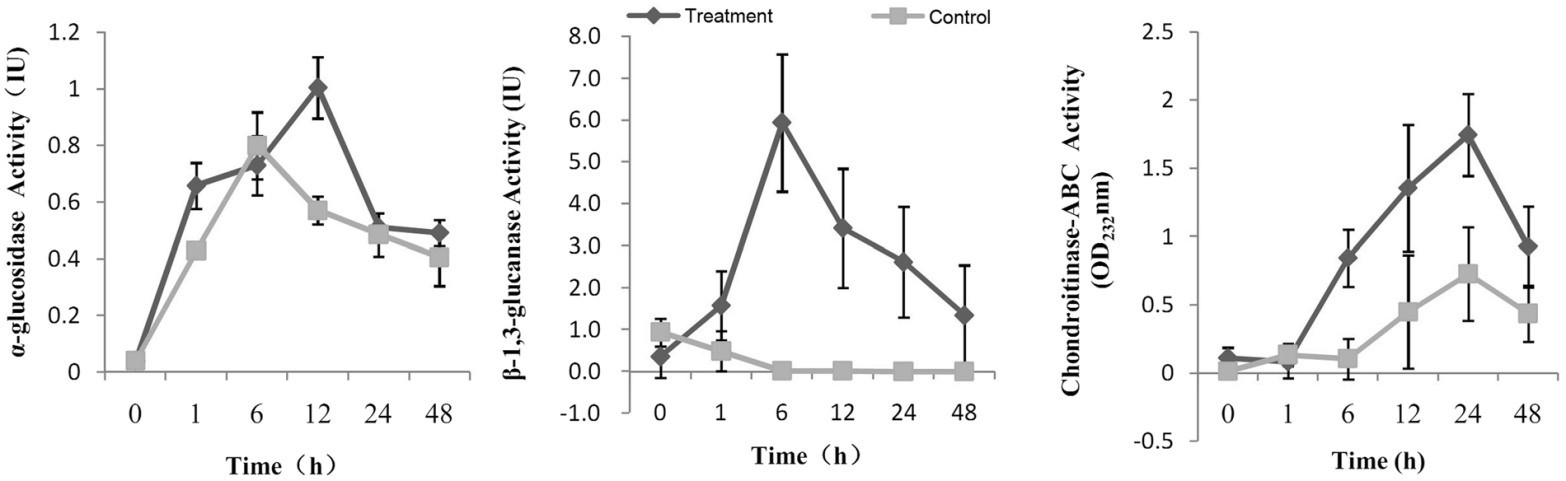
Changes of enzymatic activities of *β*-1,3-glucanase, *α*-glucosidase, and chondroitin lyase during the algicidal process. Treatment, the fungal mycelia co-cultured with algal cells; Control, the pure fungal mycelia.

As can be seen in Fig. 7, the activity of α-glucosidase in mycelia was effectively improved in treated samples. Two peaks were presented in the co-cultivated samples of 1 h and 12 h, respectively. No obvious difference was observed in 6 h, 24 h and 48 h. By contrast, the activity of *β*-1,3-glucanase was significantly increased at the first 6 h and then decreased, but the activity in mycelia with algal cells during the algicidal process was much higher than that in control. Similarly, the chondroitin lyase contents were also marked enhanced in the treated samples, which reached the highest level at 24 h. The expression patterns of these enzymes were consistent with the results of the proteomic data.

## Discussion

A small number of studies have shown that majority of the pure cultured blue algae or mixed algal cells collected from Taihu Lake (China) could be eliminated by mycelia of *T*. *abietinum* 1302BG, *T*. *versicolor* F21, *B*. *adusta* T1, *P*. *chrysosporium*, etc. Among the previously tested 60 fungal isolates, *T*. *versicolor* F21a showed the strongest algicidal ability^11^. Till date, all tested fungi with relatively strong algicidal ability belonged to saprophytic fungi^4, 7, 11^. In the *T*. *versicolor* genome, we identified 312 genes responsible for degradation polysaccharoses such as lignin and cellulose. Of these, 84 degradation enzymes were detected by proteomic analysis in this paper, with 30 of them being up-regulated in the 12 h-treated samples when compared with the 1 h samples, indicating that numerous degradation enzymes may be involved in breaking algal cells into small molecules^13, 19, 20^.

Previous studies had showed that elimination of algal cells was not attributed to antibiotics or other small chemicals. To eliminate algae by algicidal fungi, the algal cell envelop must be first broken down by enzyme(s)^13^. Lipopolysaccharides, proteins, lipids and carotenoids are the main constituents of the outer membrane of cyanobacteria^21^, and peptidoglycan constitutes the underlying layer^21, 22^. Du *et al*.^13^ believed that extracellular enzymes such as cellulase, *β*-glucosidase, protease, laccase and manganese peroxidase play important roles during the algicidal process of *T*. *versicolor* F21a^13^. In the present study, dozens of enzymes belonging to the Glycoside Hydrolases and Carbohydrate Esterases subfamilies, which can be classified as endoglycosidase and exoglycosidase, were detected during the algicidal process. Endoglycosidase may play a vital role in attacking and breaking the algal cell envelop. Protein with ID 175484 is an endo-1,3(4)-*β*-glucanase, which can cleave macromolecules such as peptidoglycan, suggesting its role in cell wall cleavage. Proteomics analysis in our study not only detected more types of extracellular enzymes (isoenzymes), but also observed intracellular enzymes that can potentially accelerate the algicidal process. Many up-regulated fungal proteins contain *α*-galactosidase (Protein ID: 159574), *β*-1,3-glucanase (Protein ID: 134935, 172368, 120979), *α*-glucosidase (Protein ID: 58033), mannosyl-oligosaccharide 1,2-*α*-mannosidase (Protein ID: 123722, 54985) and cellobiohydrolase (Protein ID: 37162), which can hydrolyze other cell wall components. Peptidase and exonuclease can degrade algal proteins and DNA respectively. Up-regulated manganese peroxidase (2 isoenzymes), cytochrome c peroxidase, glucose-methanol-choline oxidoreductase, alcohol oxidase are helpful to degrade the algal debris into fungal components (Tables S1, S3). Compared with the study of Du *et al*.^13^, additional types of degradation enzymes were detected in our investigation. It is worth noting that four up-regulated enzymes (Protein ID: 34231, 138905, 111754, 159259) belong to the Polysaccharide Lyases group. Protein with ID: 111754 is a chondroitin ABC lyase, which cleaves (1–4)-*β*-galactosaminic bonds between N-acetylgalactosamine and either D-glucuronic acid or L-iduronic acid, indicating that it can degrade peptidoglycans^23^. Alginate lyase (Protein with ID: 34231) was slightly up-regulated in the 12 h samples as compared to the 1 h samples (Tables S2, S3). Alginate or alginic acid is an anionic polysaccharide that is widely distributed in the cell walls of algae. Alginate lyase catalyzes the degradation of alginate into small molecules by a *β*-elimination reaction^23^. To our knowledge, chondroitin ABC lyase and alginate lyase have been identified for the first time in the present study by proteomics techniques as components of key decomposition enzymes during the algicidal process.

Algal cells can be decomposed into small molecules such as N-acetylglucosamine, N-acetylmuramic acid, glucose, amino acids, purines and pyrimidines. To utilize the small molecules from the degraded cells, the molecules must be assimilated into fungal cells by transporters via energy consumption^24–26^. Some of the molecules can be directly used as substrates to synthesize macromolecules, while others may be converted into other molecules for further utilization by fungal mycelia. Carbon metabolism, selenocompound metabolism, sulfur assimilation and metabolism, and several amino acid biosynthesis pathways were enriched, indicating that they play vital roles in converting small molecules into fungal components (Table 1, Fig. 3d). In particular, pyruvate decarboxylase (EC 4.1.1.1), alcohol dehydrogenase (EC 1.1.1.1), aldehyde dehydrogenase (EC 1.2.1.3), acetyl-CoA synthetase (EC 6.2.1.1) and thioltransacetylase A (EC 2.3.1.12) of the pyruvate metabolism pathway were up-regulated (Fig. S1)^18^. Proteins related to the tricarboxylic acid cycle were also obviously up-regulated (Table S3, Fig. S2)^16–18^. Pyruvate carboxylase (EC 6.4.1.1) catalyzes pyruvate, ATP and HCO^3−^ to generate oxaloacetate, which is a component of TCA. Succinate dehydrogenase (EC 1.3.5.1) and succinate-CoA ligase (EC 6.2.1.4 and EC 6.2.1.5) of TCA were up-regulated, suggesting that a large amount of ATP may be produced. As the “molecular unit of currency”^27^, ATP can be utilized in the transportation of small molecules or participates in the synthesis of macromolecules. Our results indicated that pyruvate metabolism and TCA play a critical role in response to a nutrient poor environment by increasing energy production in the early stages of degradation. Valine, leucine, isoleucine, aspartate and cysteine may be further synthesized (Fig. S3) owing to the difference in amino acid composition between blue algae and fungal mycelia^17, 18^. The amounts of these amino acids are not sufficient for direct utilization by the fungus, and may be synthesized further in the fungal mycelia.

Proteomic evidence in this study suggested that the disappearance of living algal cells and the increase in fungal biomass could be attributed to a combination of roles of different degradation enzymes, transporters and several key metabolic pathways. The molecular mechanism for the elimination of complex algal cells by fungal mycelia was far more elaborate than the degradation of a single chemical compound. Previous investigation has shown that *T*. *versicolor* F21a can quickly and completely degrade co-cultivated algal cells. To eliminate the algal cells, *T*. *versicolor* F21a utilizes hundreds of significantly differentially regulated proteins that degrade, transport and convert different algal components into components of the fungal mycelia. Further work is needed to reveal changes and functions of different degradation enzymes during the entire algicidal process of fungi in this new mode.

## Materials and Methods

### Fungal strain and its cultivation

The fungus *T*. *versicolor* F21a was isolated from the soil of Zijin maintain (Nanjing, China) as described by Han *et al*.^11^. A piece of round fungal mycelium (7 mm) from the edges of a plate was inoculated in a 9 cm plate containing 15 mL of potato medium (4 g·L^−1^ potato starch, 20 g·L^−1^ dextrose, and 15 g·L^−1^ agar) under static conditions (28 °C). After 5 d of cultivation, the fungal mycelia were used for inoculation in algicidal experiments.

### Algal strain and its cultivation

The strain *Microcystis aeruginosa* PCC7806 was provided by the Freshwater Algae Culture Collection of the Chinese Academy of Sciences (Wuhan, China) and was propagated in BG11 medium^11^. The strain was kept at 25 °C in an illumination incubator under a 12:12 h (light/dark) cycle at approximately 90 μmol photons m^−2^ s^−1^ provided by cool white fluorescent lamps.

### Co-cultivation of fungus and algal cells

Batch liquid tests were conducted in 250 mL Erlenmeyer flasks containing 100 mL of algal medium and a fungal inoculum as described by Han *et al*.^11^. Briefly, the prepared fungal mycelia were transferred into a flask and co-cultivated in a shaking incubator (25 ± 2 °C, 125 rpm). Co-cultivated fungal mycelia were sampled after 1 h and 12 h. With the consideration of the fungal mycelia need a short time to adjust in the new environment, 1 h samples were used as the initial samples. All experiments were performed in triplicate.

Total chlorophyll-a was extracted with 90% acetone and estimated according to the Standard Methods for the Examination of Water and Wastewater (1998).

### Protein extraction, digestion and TMT labeling

Co-cultivation samples of fungal mycelia and algal cells were ground to a powder in liquid nitrogen and three biological replicates of each were prepared for proteomic analyses. The cell powder was resuspended in lysis buffer (8 M urea, 10 mM DTT, 2 mM EDTA and 1% protease inhibitor Cocktail VI) and sonicated thrice on ice. The debris was removed by centrifugation at 20,000 g for 10 min and the remaining protein was precipitated with pre-cooled 15% TCA for 2 h at −20 °C. After centrifugation, the precipitate was washed thrice with cold acetone and redissolved in buffer (8 M urea, 100 mM TEAB, pH 8.0). A 2-D Quant kit (GE Healthcare, USA) was used to determine protein concentrations. The protein solution was digested with trypsin (Promega) at 1:50 trypsin-to-protein mass ratio for the first overnight digestion and 1:100 trypsin-to-protein mass ratio for a second 4 h-digestion. Approximately 100 μg protein from each sample was digested with trypsin for further experiments. After trypsin digestion, the peptide mix was desalted by Strata X C18 SPE column (Phenomenex) and vacuum-dried. The dried peptide mix was reconstituted in 0.5 M TEAB and processed as per the manufacturer’s protocol by the 6-plex TMT kit. Briefly, one unit of TMT reagent (defined as the amount of reagent required to label 100 μg of protein) was thawed and reconstituted in 24 μl ACN. The peptide mixtures were then incubated for 2 h at room temperature and pooled, desalted and dried by vacuum centrifugation.

### HPLC fractionation

The labeled peptides were fractionated by high pH reverse-phase HPLC using Agilent 300 Extend C18 columns (5 μm particles, 4.6 mm ID, 250 mm length). Briefly, the peptides were initially separated on a 2% to 60% acetonitrile gradient in 10 mM ammonium bicarbonate at pH 10 over 80 min into 80 fractions. The peptides were then combined as per the manufacturer’s protocol for LC-MS/MS analysis.

### Quantitative proteomic analysis by LC-MS/MS

#### LC-MS/MS Analysis

The labeled peptides were dissolved in 0.1% FA (Formic acid) and directly loaded onto a reversed-phase pre-column (Acclaim PepMap 100, Thermo Fisher Scientific). Peptide separation was performed in a reversed-phase analytical column (Acclaim PepMap RSLC, Thermo Scientific). The gradient was comprised of an increase from 6% to 22% of solvent B (0.1% FA in 98% acetonitrile) over 26 min, then 22% to 35% in 8 min and increasing to 80% in 3 min, then holding at 80% for the last 3 min, at a constant flow rate of 350 nl min^−1^ on an EASY-nLC 1000 UPLC system. The resulting peptides were analyzed on a Q ExactiveTM hybrid quadrupole-Orbitrap mass spectrometer (Thermo Fisher Scientific).

#### Database search

Data were deposited in the PRIDE database^28^ with the identifier PXD005568. The Mascot search engine (v.2.3.0) was employed to process MS/MS data. Tandem mass spectra were searched against 14296 predicted protein sequences *T*. *versicolor* FP-101664 SS1 that were downloaded from the fungal genomics resource at JGI (v1.0, 2011-01-05). For peptide search, Trypsin/P was specified as the cleavage enzyme allowing up to 2 missing cleavages. Mass error was set to 10 ppm for precursor ions and 0.02 Da for fragment ions. Carbamidomethyl on Cys, TMT-6Plex on peptides N-term and Lys were specified as the fixed modification. Oxidation on Met was specified as the variable modification. For protein identification, only peptides with scores significant at the 99% confidence level and peptides ion score was set as $\geqq$20 were considered to be reliable. Only proteins with significant quantitative ratios between the two treatments (*p* < 0.05) and with fold changes >1.2 or <0.83 were considered to be differentially expressed^15^.

### Protein annotation and enrichment analysis

All identified proteins were annotated based on the UniPort-GOA database, Gene Ontology database, InterProScan database, dbCAN and KEGG Pathway database. The GO function of the proteins was classified into the following three categories: biological process, cellular component and molecular function. The InterPro domain database was used to annotate protein domain function based on protein sequence alignment method. WoLF PSORT was used to predict subcellular localizations of proteins in cells. Fisher’s exact test was used to obtain the enriched functional terms. Categories with P value < 0.05 were considered as enriched.

### Statistical analysis

The proteins quantified in this study were divided into four quantitative categories based on the quantification ratio: Q1 (0 < Ratio L/H < 0.67), Q2 (0.67 < Ratio L/H < 0.83), Q3 (1.2 < Ratio L/H < 1.5) and Q4 (Ratio L/H > 1.5)^15, 16^. Quantitative category based clustering was performed. Q1, Q2, Q3 and Q4 indicated different L/H ratios in the quantification results. Geometric mean of three biological replicates was calculated for each sample and then the ratio was calculated accordingly; P value was calculated from 2-sample t-test.

Clustering Method: All substrate categories obtained after enrichment were collated along with their P values, and then filtered for those categories that were enriched in at least one of the clusters with P value < 0.05. This filtered P value matrix was transformed by the function x = −log_10_(P value). The x values obtained were z-transformed for each category. These z scores were then clustered by one-way hierarchical clustering (Euclidean distance, average linkage clustering) in Genesis. Cluster membership was visualized by a heat map using the “heatmap.2” function from the “gplots” R-package.

### Assay of enzymatic activities

Three significantly up-regulated decomposition enzymes were selected to test their activities. Fungal mycelia with or without algal cells during the entire algicidal process (0 h, 1 h, 6 h, 12 h, 24 h and 48 h) were used to prepare crude enzymes according to Du *et al*.^13^. Enzymatic activity of *β*-1,3-glucanase was determined spectrophotometrically according to Jin *et al*.^29^ and Vázquez-Garcidueñas *et al*.^30^. Laminarin (Sigma) was used as the substrate and one unit of *β*-1,3-glucanase activity was defined as the amount of enzyme that catalyzed the release of 1 µg of glucose equivalents in one minute. The activity of α-glucosidase was performed following the modified method of Mohamed *et al*.^31^. 3,4-Nitrophenyl α-D-glucopyranoside (Sigma) was used as the substrate. One unit of α-glucosidase activity was defined as the amount of enzyme that catalyzed the release of 1 µmol of pNP equivalents in one minute. Chondroitin lyase was assayed as per the modified method described previously^32, 33^. Chondroitin sulfate (sigma) was used as the substrate to demonstrate differences between the treatments and controls.

## Conclusions

In this study, 3,754 proteins of *T*. *versicolor* F21a were identified during the algicidal process. Among them, 2,809 unique proteins could be quantified. 30 proteins with biomass degradation capacities were significantly up-regulated during the process. ~10% of the up-regulated proteins were extracellular enzymes. The major metabolic pathways identified were carbon metabolism, selenocompound metabolism, sulfur assimilation and metabolism, along with several amino acid biosynthesis pathways.

## Electronic supplementary material

Figure S1-S3_Table S1-S3
