# Supplementary material for: Proteomic analysis reveals large amounts of decomposition enzymes and major metabolic pathways involved in algicidal process of Trametes versicolor F21a
Source: Sci Rep. 2017 Jun 20;7:3907. doi: 10.1038/s41598-017-04251-1 (PMC5478636; doi:10.1038/s41598-017-04251-1)
Supplement: Supplementary file 1 — Figure S1-S3_Table S1-S3 [file 41598_2017_4251_MOESM1_ESM.pdf]

manuscript title: Proteomic analysis reveals large amounts of decomposition enzymes and major metabolic pathways involved in algicidal process of *Trametes versicolor* F21a

author list: Xueyan Gao[1,†], Congyan Wang[2,†], Wei Dai[1], Shenrong Ren[1], Fang Tao[1], Xingbing He[3], Guomin Han[1,*], Wei Wang[1,*]

Figure S1. The significantly differentially regulated proteins in the pyruvate metabolism pathway. Red, up-regulated proteins; Green, down-regulated proteins.

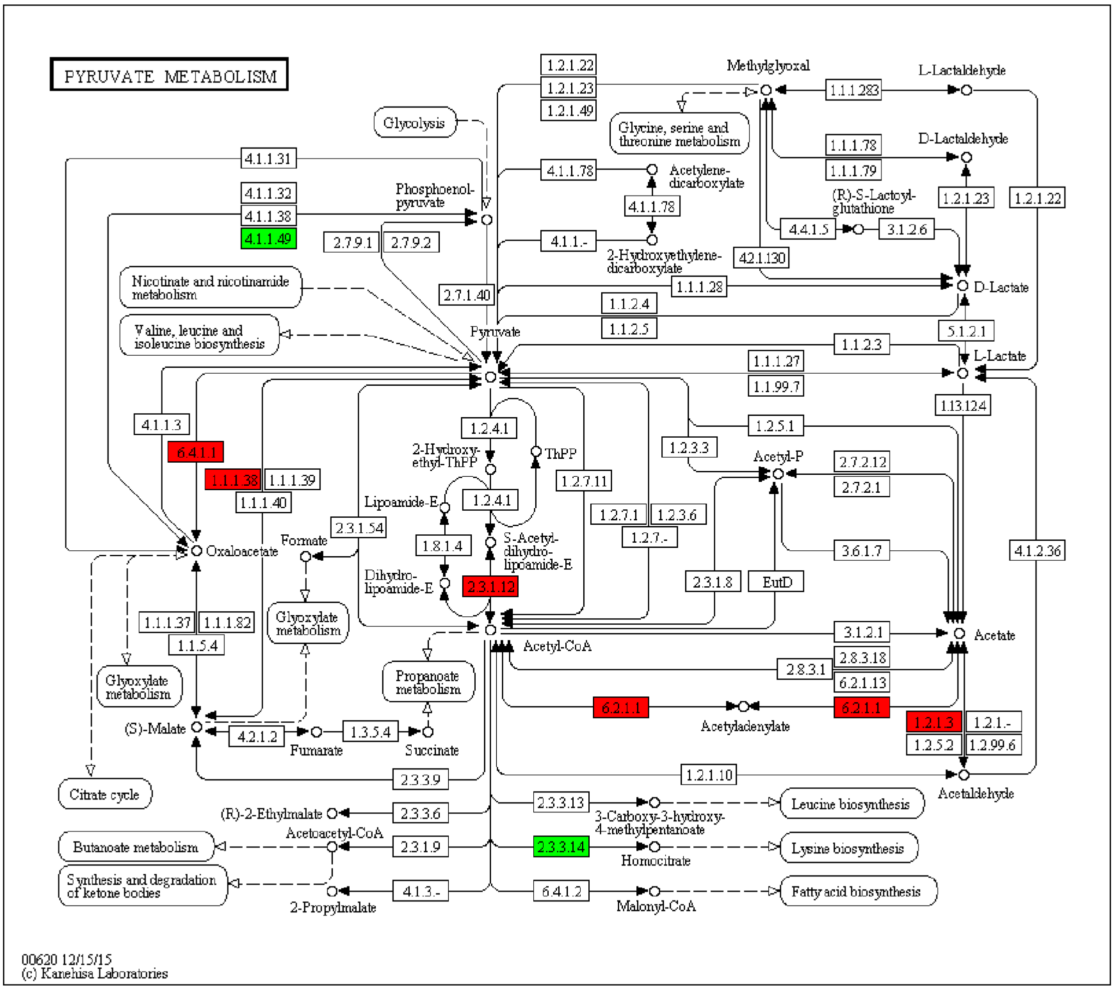

Pyruvate metabolism (source-http://www.kegg.jp/kegg/kegg1.html)

Figure S2. The significantly differentially regulated proteins in the tricarboxylic acid cycle pathway. Red, up-regulated proteins; Green, down-regulated proteins.

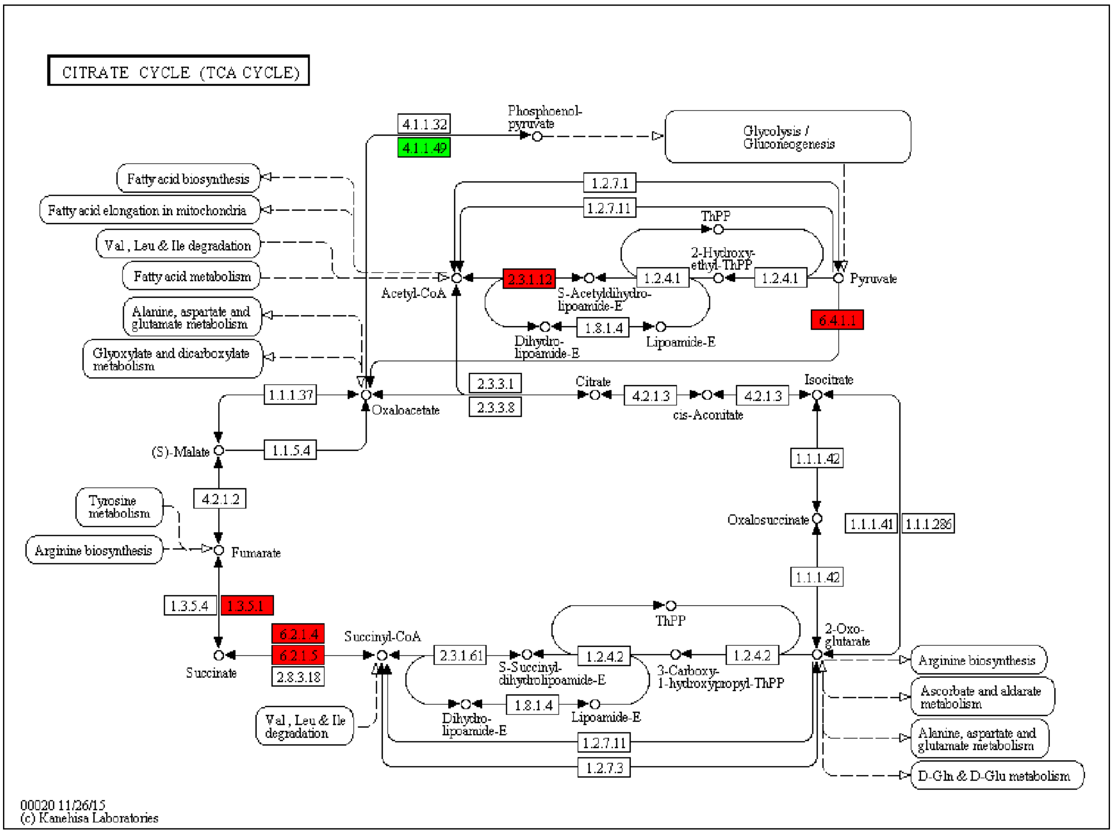

TCA cycle (source-http://www.kegg.jp/kegg/kegg1.html)

Figure S3. Amino acid biosynthesis pathways involved in the algicidal process.

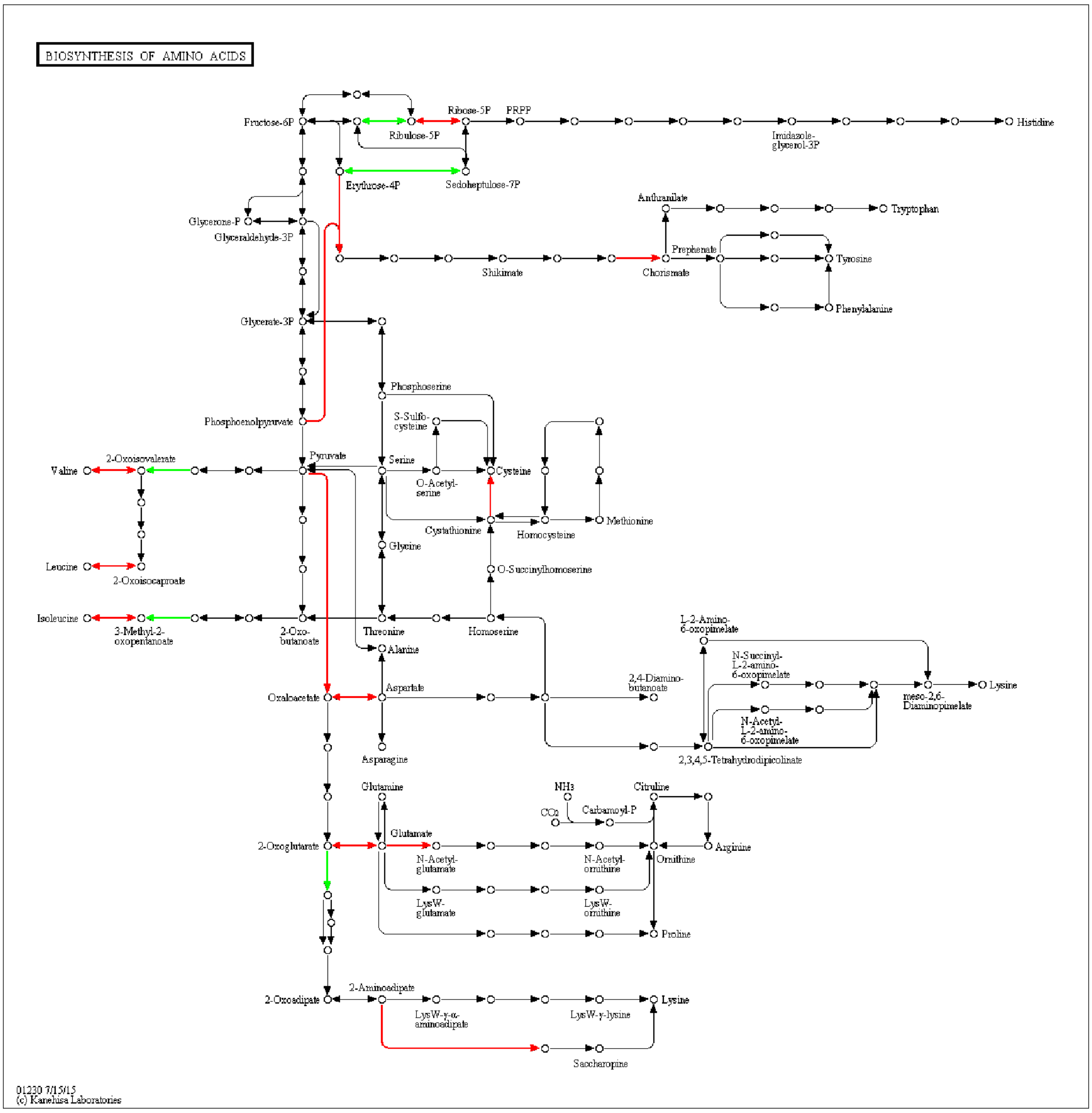

Biosynthesis of amino acids (source-http://www.kegg.jp/kegg/kegg1.html)

Table S1. The classification of detected decomposition enzymes of *T. versicolor* F21a. GH, Glycoside Hydrolases; AA, Auxiliary Activities; CE, Carbohydrate Esterases; PL, Polysaccharide Lyases.

Enzyme Classes	Enzyme SubClasses	No. of Decomposition Enzymes in Genome	No. of Detected Decomposition Enzymes
Auxiliary Activities	AA2	27	4
	AA3	23	9
	AA5	9	3
	AA8	2	0
	AA9	18	0
Carbohydrate Esterases	CE1	26	14
	CE12	2	2
	CE4	5	2
	CE8	2	0
Glycoside Hydrolases	GH1	2	1
	GH10	6	0
	GH115	2	1
	GH12	5	0
	GH128	4	0
	GH13	6	3
	GH15	4	1
	GH16	29	4
	GH17	1	1
	GH18	20	2
	GH2	5	1
	GH27	3	1
	GH28	9	1
	GH3	12	6
	GH30	4	0
	GH31	5	4
	GH32	3	1
	GH35	2	1
	GH43	3	0
	GH45	1	0
	GH47	5	4
	GH5	22	8
	GH51	2	0
	GH53	1	0
	GH55	2	1
	GH6	1	0
	GH7	4	0

	GH71	5	0
	GH74	2	1
	GH78	4	0
	GH79	11	1
	GH85	1	1
	GH9	1	0
Polysaccharide Lyases	PL14	6	2
	PL15	2	2
	PL4	1	0
	PL8	2	2
	Total	312	84

Table S2. Changes in expression of detected decomposition enzyme genes. GH, Glycoside Hydrolases; AA, Auxiliary Activities; CE, Carbohydrate Esterases; PL, Polysaccharide Lyases.

Protein accession	Protein description	T12/T1 Ratio	Regulated Type	P value
26239	AA2	1.47		0.286007
26601	AA2	1.329	up	0.005797
44897	AA2	3.702	up	0.004783
74595	AA2			
130619	AA3			
134467	AA3	0.991		0.931577
167157	AA3			
174536	AA3	1.168		0.289844
174721	AA3	1.264		0.233810
176148	AA3	0.876		0.358453
57372	AA3	1.116		0.388914
59756	AA3	1.011		0.895376
68834	AA3			
130016	AA5	0.959		0.844766
61229	AA5	1.95	up	0.000907
62839	AA5	0.82		0.722565
116106	CE1	1.067		0.466033
152697	CE1			
163026	CE1	0.794	down	0.048869
169347	CE1	0.953		0.737428
169905	CE1	0.951		0.814559
172632	CE1	0.896		0.126547
25474	CE1	0.826		0.068532
26004	CE1			
27033	CE1	1.218	up	0.039281
27331	CE1	0.726	down	0.001765
28938	CE1	0.812		0.235954
29674	CE1	0.919		0.431523
42128	CE1	0.824		0.112462
59093	CE1	0.82		0.067715
165799	CE12	0.874		0.047918
64135	CE12	0.916		0.019667
168111	CE4			
68340	CE4	0.808		0.070841
171192	GH1	1.187		0.424115
49304	GH115	0.884		0.381094
26884	GH13	0.961		0.775094
30260	GH13			
75497	GH13	1.223	up	0.006005
156418	GH15	1.167		0.015216

134935	GH16	2.461	up	0.004073
137261	GH16	1.065		0.409968
175484	GH16	1.261	up	0.035876
59797	GH16			
125216	GH17	0.846		0.022514
142040	GH18	0.88		0.246459
18215	GH18	0.992		0.956560
162659	GH2	0.907		0.225394
159574	GH27	1.003		0.983030
171861	GH28	0.428	down	0.026715
127171	GH3	0.822		0.237384
127375	GH3	0.858		0.071624
146818	GH3	1.092		0.347247
151588	GH3	0.826		0.241062
170938	GH3	0.915		0.269731
71035	GH3	0.952		0.656239
110860	GH31	0.961		0.705581
146103	GH31	0.9		0.138137
173291	GH31	1.167		0.050646
58033	GH31	2.145	up	0.003710
149280	GH32			
37024	GH35	0.844		0.226812
123722	GH47	1.265	up	0.002694
131501	GH47	0.515	down	0.014188
44565	GH47	0.864		0.467581
54985	GH47	1.119		0.348562
130762	GH5			
148125	GH5			
155564	GH5	0.943		0.494088
172368	GH5	1.05		0.548148
173244	GH5			
32196	GH5	1.131		0.312239
35803	GH5			
40418	GH5	0.914		0.281090
120979	GH55	1.016		0.937197
37162	GH74	1.065		0.060720
144865	GH79	0.695		0.279281
164019	GH85	0.844		0.012212
153895	PL14			
34231	PL14	1.136		0.304725
138905	PL15	1.214		0.074484
68108	PL15	0.924		0.499390
111754	PL8	6.881	up	0.001413
159259	PL8	1.06		0.669082

Table S3. Annotation of up-regulated proteins by NCBI.

Protein Id	Seq. Description
67857	Domain of unknown function DUF2235;
41381	hypothetical protein
33213	NEFA-interacting nuclear protein NIP30, N-terminal;
111550	GNAT domain; Acyl-CoA N-acyltransferase;
55649	Phospholipid/glycerol acyltransferase;
113719	SET domain; Rubisco LSMT, substrate-binding domain;
142047	AAA+ ATPase domain; ABC transporter-like;
69564	hypothetical protein
117358	Thioesterase superfamily; HotDog domain;
27033	Alpha/Beta hydrolase fold;
116434	Globin, structural domain; tetrapyrrole binding;heme binding
70135	hypothetical protein
35457	hypothetical protein
18976	Tetratricopeptide-like helical domain;
63852	Immunoglobulin E-set; Arrestin C-terminal-like domain;
19223	hypothetical protein
46817	Zn(2)-C6 fungal-type DNA-binding domain; Transcription factor domain, fungi;
28664	Zn(2)-C6 fungal-type DNA-binding domain;
124822	S-adenosyl-L-methionine-dependent methyltransferase; Helicase superfamily 1/2;Helicase;
149403	NAD(P)-binding domain;
126602	hypothetical protein
125518	hypothetical protein
72848	hypothetical protein
128184	RNA recognition motif domain; Nucleotide-binding alpha-beta plait domain;
171395	Siderophore biosynthesis protein; Acyl-CoA N-acyltransferase;
30580	hypothetical protein
171877	Ubiquitin-activating enzyme; Molybdenum cofactor biosynthesis;
50918	hypothetical protein
173577	Metallo-dependent phosphatase-like; 5'-Nucleotidase, C-terminal;
66816	hypothetical protein
154380	Translation Initiation factor eIF- 4e-like domain;
23578	hypothetical protein
74741	hypothetical protein
135027	Zinc finger, C2H2-like;
175203	Nucleotide-diphospho-sugar transferases;
157280	Beta-lactamase-like;
138083	malate dehydrogenase
109519	gram-domain-containing protein
32746	thioredoxin
157667	heme peroxidase;tetrapyrrole binding;heme binding
109478	mitochondrial carrier
32927	mitochondrial amino-acid acetyltransferase

158015	mitochondrial carrier
158017	fas1 domain-containing protein
111426	p-loop containing nucleoside triphosphate hydrolase protein
68127	triose-phosphate transporter
68131	glycine dehydrogenase
41748	succinate- ligase
33161	peroxisomal membrane protein pmp22
139094	mitochondrial carrier
139210	pyruvate dehydrogenase
68341	glycoside hydrolase family 72 protein
139674	atp-dependent dna ligase
55519	isopentenyldiphosphate isomerase
111754	polysaccharide lyase family 8 protein
109115	glutamic oxaloacetic transaminase aat1
25862	peroxisomal biogenesis factor 11
108904	branched-chain amino acid aminotransferase ii
25901	bromodomain-containing protein
55705	cysteine proteinase; peptidase activity;
159686	nucleotide-binding protein;exonuclease
140224	biotin synthase
112226	heterotrimeric g protein alpha subunit 4
42537	duf21-domain-containing protein
68760	type ii dna topoisomerase
17003	chorismate synthase
111458	oxysterol-binding protein;tetrapyrrole binding;heme binding
55962	multidrug resistance-associated abc transporter
160473	h+ nucleoside cotransporter
56077	rna polymerase ii-associated protein
26376	adenylylsulfate kinase activity
26392	hypothetical protein TRAVEDRAFT_26392
113884	fumarate reductase;tetrapyrrole binding;heme binding
26433	utp-glucose-1-phosphate uridylyltransferase
56171	udp-glycosyltransferase glycogen phosphorylase
161111	small subunit of carbamoyl phosphate synthase
34338	phosphomevalonate kinase
26601	cytochrome c peroxidase;tetrapyrrole binding;heme binding
114984	gtp-binding protein 1
26633	sulfate adenylyltransferase denylylsulfate kinase activity
43618	transcription regulator
62886	endoplasmic oxidoreductin
161569	nadph-dependent d-xylose reductase
142245	endosomal p24a protein
69454	ankyrin repeat domain protein
62974	metallo-hydrolase oxidoreductase

43865	glycerol-3-phosphate 1-acyltransferase
114801	nad -binding protein
115459	golgi apparatus membrane protein tvp38
162407	altered inheritance rate of mitochondria protein 38
56675	nuclear protein
34877	stomatin family protein
34879	cysteine proteinase; peptidase activity;
162694	cleavage and polyadenylation specificity factor subunit
143570	membrane protein
117906	phosphoadenosine phosphosulfate reductase thioredoxin
163103	ap-domain-containing protein
27320	riboflavin kinase
70168	nuclear segregation protein bfr1
70243	duf1793-domain-containing protein
44897	manganese peroxidase;tetrapyrrole binding;heme binding
144642	tetrapyrrole binding;heme binding;Acyl-CoA dehydrogenase
117197	rna-binding domain-containing protein
164101	kinesin-like protein
144721	Acyl-CoA dehydrogenase
145091	aspartate aminotransferase
164956	mfs general substrate transporter
63888	fact complex subunit spt16; peptidase activity
165208	duf382-domain-containing protein
145845	adaptor protein complex beta subunit
165360	p-loop containing nucleoside triphosphate hydrolase protein
36183	rab-protein geranylgeranyltransferase
19288	fas1 domain-containing protein
165737	aaa-domain-containing protein
57912	snf2 chromatin remodeling protein
119241	gtp-binding protein ypt1
58033	glycoside hydrolase family 31 protein
46294	aldo keto reductase
166243	isocitrate lyase
147039	pyruvate decarboxylase
121918	3-deoxy-7-phosphoheptulonate synthase
166729	vacuolar protein sorting-associated protein 45
147404	ssrecog-domain-containing protein
64480	actinin-like protein
166830	nonribosomal peptide synthetase 12
36915	acyltransferase ctase cot cpt
28788	escrt-ii complex vps25 subunit
71937	cyclophilin-like protein
37283	pirin domain-containing protein
64856	von willebrand ring finger domain-containing protein

123722	glycoside hydrolase family 47 protein
58730	protein
47707	p-loop containing nucleoside triphosphate hydrolase protein
148803	hydroxymethylglutaryl- synthase
72170	membrane protein
72201	pyruvate carboxylase
168510	mfs monosaccharide transporter
168550	apses-domain-containing protein
72253	cytochrome p450;tetrapyrrole binding;heme binding
168593	dihydroxyacetone kinase
149223	mfs monosaccharide transporter
65084	transcriptional activator
29393	rho gtpase activation protein
126407	metallo-hydrolase oxidoreductase
125356	multifunctional beta-oxidation protein
59063	proteophosphoglycan ppg4
125791	zip-like iron-zinc transporter
38031	transcription factor iws1
150183	abc transporter
65423	hypothetical protein TRAVEDRAFT_65423
125068	acn9-domain-containing protein
48870	Dyp-type peroxidase;tetrapyrrole binding;heme binding
170018	duf544-domain-containing protein
59445	succinate dehydrogenase cytochrome b560 subunit
150975	cyanamide hydratase
170411	anti-silence-domain-containing protein
59641	actin-like atpase domain-containing protein
38598	mitochondrial carrier
38613	dna rna polymerase
30240	beta-lactamase transpeptidase-like protein; peptidase activity;
129002	g-alpha-domain-containing protein
73434	zf-parp-domain-containing protein
171660	acetyl- synthetase-like protein
50314	s-adenosyl-l-methionine-dependent methyltransferase
171718	tetracenomycin polyketide synthesis hydroxylase
30565	cystathionine gamma-lyase
172153	ribose-5-phosphate isomerase
172170	nadh dehydrogenase
50847	dehydrogenase e1 and transketolase domain-containing protein 1;oxoglutarate dehydrogenase
153101	band 7 domain-containing protein
153200	oligopeptide transporter
172544	endoplasmic reticulum vesicle protein 25
131096	GroES-like protein
173102	nad-dependent formate dehydrogenase

60534	iron-sulfur cluster assembly accessory protein isa2
154039	myo-inositol-1-phosphate synthase
31290	cytochrome p450;tetrapyrrole binding;heme binding
132919	cysteine partial; peptidase activity
173629	phosphoenolpyruvate pyruvate domain-containing protein
154403	swib-domain-containing protein
52389	transcription elongation factor spt5
60843	cohesin subunit psc3
74716	alpha beta-hydrolase
31575	nad-aldehyde dehydrogenase
23785	chloroperoxidase-like protein
74763	alpha beta-hydrolase; peptidase activity
154958	ubiquitin-protein ligase molybdopterin-converting factor
67110	tpr-like protein
135477	thioredoxin-like protein
61229	copper radical oxidase
134935	glycoside hydrolase family 16 protein
134691	3 -bisphosphate nucleotidase hal2
175376	phosphatases ii
156126	coproporphyrinogen iii oxidase
175484	glycoside hydrolase family 16 protein
156153	nad-specific glutamate dehydrogenase
175547	n-acetylhexosaminidase
53859	cytochrome p450;tetrapyrrole binding;heme binding
156571	thioredoxin reductase
67500	acetate-- ligase
75497	glycosyltransferase family 5 protein
156866	mitochondrial protein
32319	response regulator
54358	thioredoxin-like protein
176288	alpha beta-hydrolase
34231	alginate lyase
